# Anaerobic digestion of poultry manure to power a poultry farm in Ba: Pilot and techno-economic study

**DOI:** 10.1016/j.heliyon.2024.e36325

**Published:** 2024-08-22

**Authors:** Geeta M. Naidu, Atul Raturi, Francis S. Mani

**Affiliations:** a Master of Science Programme, School of Information Technology, Engineering, Mathematics and Physics, University of the South Pacific, Suva, Fiji; b School of Information Technology, Engineering, Mathematics and Physics, University of the South Pacific, Suva, Fiji; c School of Agriculture, Geography, Environment, Ocean and Natural Sciences, University of the South Pacific, Suva, Fiji

**Keywords:** Solid state anaerobic digestion, Poultry manure, Biogas, Renewable energy, Pacific islands

## Abstract

Although poultry is the largest meat by volume produced in Fiji, there has not been any established study, nor application of the anaerobic digestion (AD) of poultry manure (PM) in the country. This paper aims to determine the techno-economic feasibility of the AD of PM to power a poultry farm in Fiji. A pilot scale study was first conducted with mono-digestion batches of poultry manure, and co-digestions with kitchen waste (KW) and newspaper waste (NPW). Solid state anaerobic digestion (SSAD) was employed in all the batches, and the key operational parameters of AD were studied, along with its influence on biogas production. The pilot study revealed that even slight changes in environmental temperature had the greatest effect on biogas production. The most resilient to the temperature changes were the co-digested feedstocks of KW. Yet, given a substantial AD period, the anaerobes in the mono-digesters were able to eventually acclimatize to the SSAD environment, and produce the overall highest biogas production. The pilot study results were then used to conduct a feasibility study of the full-scale design. The analysis showed that the SSAD system would generate 189.46 MWh of electricity annually, with a levelized cost of energy of FJ$0.17/KWh.

## 1 Introduction

With the current global population reaching more than 8 million people [1], the global energy demand is expected to grow by 47 % in 2050 [2]. However, due to the current climate crisis, there is not just a need for energy, but “affordable and clean energy”, as stated under goal seven (SDG7) of the United Nations Sustainable Development Goals [3]. Fiji is a member of the Small Island Developing States (SIDS), a group of countries which are most vulnerable to the global warming effects, but also contribute the least to it. The country nonetheless endeavours to combat the climate change crisis, by currently employing approximately 59 % of renewable energy generation in its electricity profile [4].

The single largest meat volume produced in Fiji is poultry [5]. There are more than 893,000 chickens (mainly layer and broiler species) housed across Fiji's major commercial poultry farms alone, which means more than 81 tonnes of poultry manure (PM) is being produced annually in the country [6]. Yet, to the best of the author's knowledge, there has not been any study nor application of the anaerobic digestion (AD) of PM in the country. AD is one of the proven ways to utilize biowaste to produce useful energy, whilst at the same time implement an effective waste management system. It is a series of biochemical processes that uses a diverse population of bacteria to break down organic matter in the absence of oxygen, generating biogas, comprising mostly of methane (approx. 60 %) and carbon dioxide (approx. 40 %), with traces of hydrogen sulphide and water [7]. The process also produces a nutrient rich solid/liquid mixture called effluent (or digestate), which can be utilized as a soil fertilizer or conditioner [8].

At present, the PM in the country is only being sold as fertilizer or utilized for broiler beds, while the excess is stored, usually in the farm sheds itself or spread directly on land. This practice is hazardous to the environment as it can cause the spread of pathogens; In addition, over fertilization can induce excess nutrients in soil, which can lead to eutrophication of water bodies [9]. Studies have shown that pathogens in PM are significantly reduced during AD, whilst still maintaining its rich nutrients, making it a safer alternative to its raw manure as a soil conditioner or fertilizer [9].

The biogas produced from AD can be used to replace liquified petroleum gas (LPG) in engines to produce electricity, or more significantly, as cooking fuel in stoves. As of 2018, approximately 51 % of Fiji's population still relied on unclean cooking technology and fuel [10]. There is an increasing shift to cleaner fuels at a population access rate of 1.7 % per year [10]; moreover, employing AD applications for biogas production would further contribute to this cause. This would also be beneficial to the country as it spends more than FJD 550 million annually on imported fossil fuel [11]. Additionally, approximately 65 % of Fiji's renewable energy mix is from hydropower alone [4]. Thus, employing applications of AD of PM in Fiji would not only contribute to the global mitigation efforts towards the current climate crisis, but also increases the country's energy security.

Although AD is a mature technology, PM as its feedstock have been rarely used to the extent that other livestock wastes have globally, and especially in the Pacific. This is mainly due to its heterogenous nature, high nitrogen and solids content, which tend to have inhibitory effects during the digestion process [12]. Consequently, solid state (SSAD) or high solids anaerobic digestion (HSAD; more commonly known as dry anaerobic digestion), of PM have been rare due to this inherent nature. An AD process can be classified as wet, semi solid, or solid state, depending on the total solids (TS) content present in the feedstock, i.e., TS < 10 %, 10–15 %, or TS > 15 %, respectively [13]. Studies have shown that in general, TS levels and biogas production tend to have an inversely proportional relationship [14]. However, studies have also shown that if provided with a longer hydraulic retention time (HRT), the bacteria in a biodigester are able to eventually acclimatize to the high TS levels and produce substantial volumes of biogas [15]. Furthermore, the industrial-scale applications of the AD of poultry manure in other countries have also proven its viability [16].

The aim of this work is to determine the feasibility of the anaerobic digestion of poultry manure for a potential biogas powered poultry farm in Fiji. The main objectives are to firstly carry out a pilot scale study to evaluate the AD of PM and quantify the biogas that is produced from PM, in combination with other biomasses, identify the impacts of different operational parameters on the biogas production, investigate the potential electricity generation using the produced biogas and finally, perform a techno-economic feasibility analysis of the proposed full scale bioenergy system.

## 2 Materials and methods

### 2.1 Poultry farm site

The farm whereby the study was conducted is called *Nidoos No.1 Suppliers*, and is located in Ba, Fiji (site location: 17.53762, 177.66916). It houses 10,000-layer chickens belonging to the *shaver brown* breed. These birds are primarily bred for egg production and the chickens are then sold off after reaching a certain age (18–20 months following the purchase of chicks).

### 2.2 Construction of anaerobic digesters

**Fig. 1:**
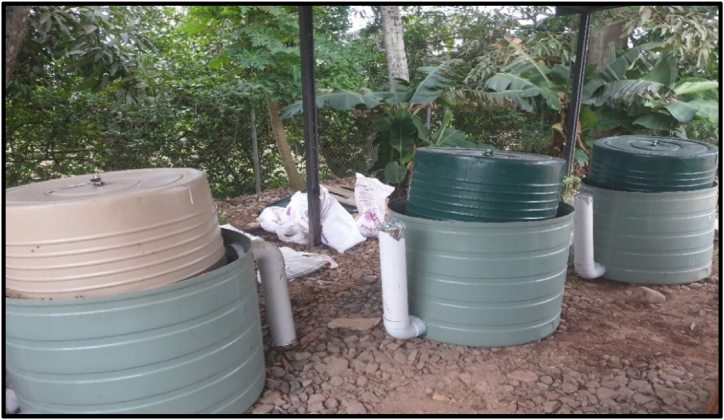
Anaerobic digesters.

**Fig. 2:**
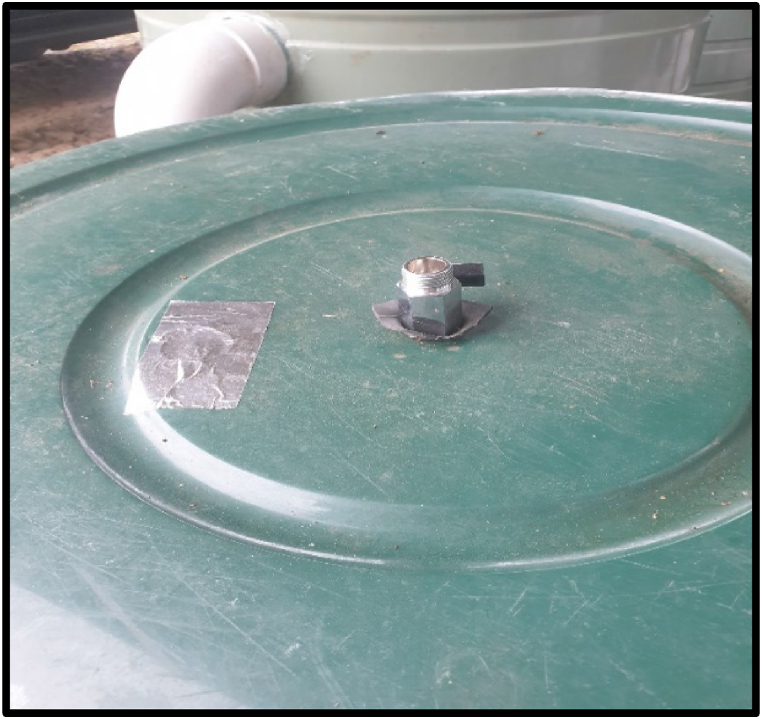
Gas holder top construction.

For the pilot study, a sample commercial biodigester of floating tank design was used as a model to build three identical digesters, whereby 500 L polyethylene tanks were used as the anaerobic reactors, and 300 L tanks were used as biogas holders (see Fig. 1). On top of the biogas holder tanks, gas valves were fitted with a recycled rubber material for an airtight seal. A small hole (diameter = 4 cm) was also drilled on top of these gas holders for inserting the pH/temperature and biogas probes, which were temporarily sealed with an aluminium foil tape in between readings (see Fig. 2). All digesters were placed under a shed, with the closest building (feed storage house) approximately 50 m away.

### 2.3 Feedstock preparation and AD operation

Fresh PM was collected from the farm a day before the commencement of an AD batch. Essentially, because past studies have shown that volatile content greatly reduces as lag period increases between feedstock collection and AD [17]. The fresh PM was mostly pure manure with no mixture of sawdust. However, it did include fragments of chicken feathers. The cow manure utilized as inoculate, was obtained from privately owned cattle, while the kitchen waste (KW) and newspaper waste (NPW) were acquired from a nearby household. The KW comprised of mostly fruit and vegetable peelings. Both the NPW and KW underwent mechanical pre-treatment only; The NPW was shredded to approximately two cm^2^ pieces, while the KW was mashed up until a slurry consistency was achieved.

Two batches of AD were conducted, with digesters 1, 2 and 3 occurring simultaneously in the first batch, and digesters 4, 5 and 6 in the second batch. Digesters 1 and 2 functioned as mono-digesters, containing poultry manure only, digesters 3 and 4 contained PM and KW, while digester 5 and 6 contained PM and NPW. Table 1 further shows the specific percentage and volume used up by the different substrates.

**Table 1:** Feedstock labelling and composition.

Digester Labelling	Substrate	Composition (%)	Volume (L)
Digesters 1 & 2	Poultry Manure	100	280
Digesters 3 & 4	Poultry manure	75	210
	Kitchen waste	25	70
Digesters 5 & 6	Poultry manure	90	252
	Newspaper waste	10	28

Out of the total 500 L digester tank, 70 % or 350 L were used as an active reactor. Twenty percent of this active volume was filled up with cow slurry inoculum. As utilized by previous studies [18,19], the dilution ratio of the cow slurry was 1:1 in this study also. This inoculum volume was only used in the first batch of AD. The inoculum for the second batch had half of its inoculum made up of cow slurry, while the other half was obtained from the effluent of the mono-digesters in the first batch. The remaining active volume (280 L) of the digester was filled up with the main substrates under investigation as shown in Table 1. Digesters 1 and 2 functioned as mono-digesters and had PM occupying its total active volume. For digesters 3 and 4, the percentage composition of PM was 75 % against 25 % of KW, while for digesters 5 and 6, PM made up 90 % of the active volume with NPW making up the remaining 10 %. All feedstocks were diluted with a substrate to water ratio of 1:2 and weighed accordingly.

With the three biodigesters that were built, two batches of AD were run, batch ne followed by batch two. Anaerobic digestion for batch one, which included digesters 1, 2, and 3, occurred simultaneously with a hydraulic retention time (HRT) of 45 days. The digesters were then reused in batch two, labelled as digesters 4, 5, and 6, operating for the same HRT.

### 2.4 Operational measurements

Temperature and pH readings were recorded daily for the first two weeks then on a weekly basis for the rest of the digestion period. A *Hanna HI8314* model pH meter was used, which was calibrated before every measurement accordingly, to the manufacturer's guide [20]. However, the pH and temperature readings for the second batch of feedstocks were measured for only the first half of its digestion period due to technical hitches. Additionally, since the digesters would be well into its stabilized stages at that point in time, it would highly unlikely affect the results of the study.

The biogas content was measured with a *Geotech Biogas 5000* analyser daily unless the occurrence of harsh weather such as a tropical cyclone or heavy rain. The biogas volume was measured with outlines made on the biogas holders, with an uncertainty of 10 L. As the biogas was collected, which caused the tank to rise, the readings were noted from the biogas holders. After the readings were taken, the gas was collected in a biogas storage bag.

The C/N ratio of the different feedstocks was computed using a comprehensive online calculator [21], according to the mass of each substrate component. The calculator was created by compiling other reliable and scientific literature, however, the C/N values generated may not be the exact amount as the results are based on average figures. Previous researchers that have measured C/N ratio utilized various elemental analysers such as the Vario MICRO Cube [12] or the LECO analyser [14], while others measured the elements with an inductively coupled plasma mass spectrometry [22]. However, employing such methods were out of the scope of the present study.

### 2.5 Physical analysis

Volatile solids suspension (VSS) test was carried for samples collected from all the digesters at the beginning and at the end of the digestion periods, i.e., two samples for each digester. The VSS test was conducted according to the American Public Health Association (APHA) standard methods [23] as carried out by many researchers [12,24,25].

### 2.6 Full scale design

The results obtained from the pilot study were then used to design a full-scale anaerobic digestion system. The calculations and steps utilized to determine the potential biogas production and electricity usage were obtained from *Anaerobic Digestion of Biowaste in Developing Countries* by Ref. [8].

#### 2.6.1 Daily feedstock availability and digester size

The mass of poultry manure, hence, the available feedstock that is produced daily by the *shaver brown* breed was calculated using:

(Equation 1) $$M_{raw}=M_{chicken}\;x\;n$$

where:

• $M_{\text{raw}}=\text{mass}\;\text{of}\;\text{raw}\;\text{feedstock}\;\left(\text{kg}\right)$.
•$M_{\text{chicken}}=\text{mass}\;\text{of}\;\text{manure}\;\text{produced}\;\text{by}\;\text{one}\;\text{chicken}\;\text{in}\;a\;\text{day}\;\left(\text{kg}/\text{day}\right)$.
•n=numberofchickens.

Also, the total available daily mass of the raw feedstock, i.e., the substrate flowrate, taking into consideration the water required for dilution, was calculated as:

(Equation 2) $$Q=M_{raw}+M_{water}$$

where:

•Q=substrateflowrate(kg/day).
•$M_{\text{water}}=\text{mass}\;\text{of}\;\text{water}\;\left(\text{kg}\;\text{or}\;L\right)$.

Hence, the total active volume of the digester was computed using:

(Equation 3) V=HRTxQ

where:

•V=totalactivedigestervolume(L).
•Q=massofdailyavailablefeedstock(kg/day).
•HRT=hydraulicretentiontime(days).

#### 2.6.2 Operational parameters

To calculate the mass of volatile solids in the feedstock from the previously calculated percentage VS, the following was used:

(Equation 4) $${VS}_{feedstock}=VS\;x\;V_{TS}$$

where:

•${\text{VS}}_{\text{feedstock}}=\text{mass}\;\text{of}\;\text{volatile}\;\text{solids}\;\text{in}\;\text{the}\;\text{feedstock}\;\left(\text{kg}\right)$.
•VS=volatilesolidscontentinfeedstock(%).
•$V_{\text{TS}}=\text{mass}\;\text{of}\;\text{total}\;\text{solids}\;\text{in}\;\text{the}\;\text{feedstock}\;\left(\text{kg}\right)$.

##### 2.6.2.1 Substrate inflow

The concentration of feedstock available daily with respect to its volatile solids content was calculated using:

(Equation 5) $$S=\frac{{VS}_{feedstock}}{V}$$

where:

•$S=\text{substrate}\;\text{concentration}\;\text{in}\;\text{the}\;\text{inflow}\;\left(\text{kgVS}m^{-3}\right)$.
•$V=\text{reactor}\;\text{volume}\;\left(m^{3}\right)$.

##### 2.6.2.2 Organic loading rate

The amount of feedstock that would be loaded daily into the digester with respect to its volatile solids content was calculated as below:

(Equation 6) $$OLR=\frac{Q\;x\;S}{V}$$

where:

•$\text{OLR}=\text{organic}\;\text{loading}\;\text{rate}\;\left(\text{kgVS}/m^{3}\text{reactor}\;\text{volume}\right)$.
•$Q=\text{substrate}\;\text{flowrate}\;\left(m^{3}/\text{day}\right)$.

#### 2.6.3 Biogas production

To calculate the volume of biogas that would be produced per day, the following equation was utilized:

(Equation 7) $$Q_{biogas}=OLR\;x\;BGY\;x\;V$$

where:

•$Q_{\text{biogas}}=\text{biogas}\;\text{flow}\;\text{rate}\;\left(m^{3}/\text{day}\right)$.
•$\text{BGY}=\text{biogas}\;\text{yield}\;\left(m^{3}/\text{kgVS}\right)$.

#### 2.6.4 Energy output

According to Ref. [8], 1 m^3^ of biogas with an average methane content of 55–60 % has an energy content of 6 kWh. Hence, the daily energy output of the biogas digester can be calculated by:

(Equation 8) $$E_{biogas}=Q_{biogas}\;x\;6\;kWh$$

where:

•$E_{\text{biogas}}=\text{electrical}\;\text{energy}\;\text{output}\;\text{of}\;\text{biogas}\;\left(\text{kWh}\right)$.
•$Q_{\text{biogas}}=\text{biogas}\;\text{flowrate}\;\left(m^{3}/\text{day}\right)$.

Thus, taking into consideration the efficiency of its engine, the actual electrical energy output from a biogas generator was computed using:

(Equation 9) $$E_{electricity}=E_{biogas\;}\;x\;\eta$$

where:

•$E_{\text{electricity}}=\text{useable}\;\text{electricity}\;\text{output}\;\text{from}\;\text{biogas}\;\text{generator}\;\left(\text{kWh}\right)$.
•$E_{\text{biogas}}=\text{energy}\;\text{output}\;\text{of}\;\text{biogas}\;\left(\text{kWh}\right)$.
•η=electricialefficiencyofbiogasengine.

Similarly, the above equation can be also used to find the heat energy ($E_{\text{heat}}$) from the CHP engine by simply substituting the heat efficiency in place of the electrical efficiency, η.

The heat energy supplied to the effluent can be used to calculate its increase in temperature by using:

(Equation 10) $$E_{heat}=m_{w}c_{w}\Delta T+m_{pm}c_{pm}\Delta T$$

where:

•$E_{\text{heat}}=\text{heat}\;\text{energy}\;\text{released}\;\text{from}\;\text{the}\;\text{CHP}\;\text{engine}$ (J)
•$m_{w}=\text{mass}\;\text{of}\;\text{water}\;\left(\text{kg}\right)$.
•$c_{w}=\text{specific}\;\text{heat}\;\text{capacity}\;\text{of}\;\text{water}\;\left(J/\text{kg}{}^{\circ}C\right)$.
•$m_{\text{pm}}=\text{mass}\;\text{of}\;\text{poultry}\;\text{manure}\;\left(\text{kg}\right)$.
•$c_{\text{pm}}=\text{specific}\;\text{heat}\;\text{capacity}\;\text{of}\;\text{poultry}\;\text{manure}\;\left(J/\text{kg}{}^{\circ}C\right)$.
•ΔT=changeintemperature(°C).

#### 2.6.5 Economic feasibility

Simple payback (SPB) time and levelized cost of energy (LCOE) were used for the economic analysis of the proposed full-scale project to indicate its viability:

(Equation 11) $$SPB\;\left(years\right)=\frac{\sum \left(Investment\;costs\right)}{\sum \left(yearly\;benefits-yearly\;costs\right)}$$

(Equation 12) LCOE(FJ$/kWh)=∑costsno.ofyearsAnnualyield(kWh)

## 3 Results and discussion

### 3.1 C/N ratio

**Fig. 3:**
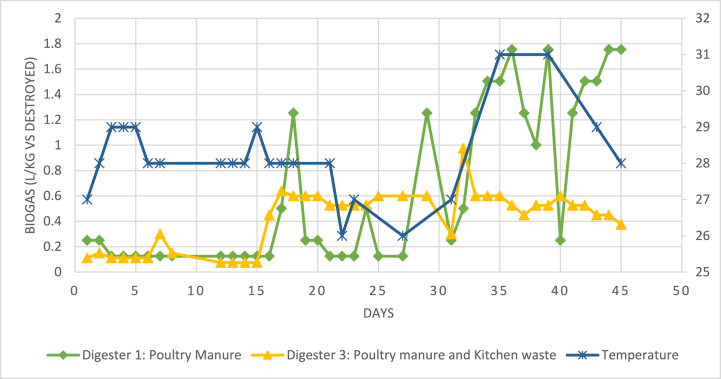
Temperature variation against biogas yield for digesters 1 and 3.

Table 1 shows the C/N values for the different feedstock and its average biogas production. Note that the average biogas production values of digesters 3 and 4 are recorded separately under Table 1; This has been done for accuracy purposes, as both the digesters occurred at separate times, hence, undergoing varying environmental conditions, despite containing the same feedstock. The most consistent biogas yield was produced by the co-digested waste of PM with KW (also see Fig. 3). This may be contributed to its C/N value being the closest to the ideal range of 15–25, for PM co-digested with agro-industrial waste, as suggested by de Oliveira Paranhos, Adarme [24]. A higher C/N ratio would cause the nitrogen to be consumed first by the methanogens, leaving behind carbon, and thus lower biogas production; While in the case of a lower C/N ratio, ammonia inhibition would occur, and hence, reduce biogas yield also [17]. This trend was also observed during the pilot scale study, whereby, the co-digested feedstock of PM with NPW, that had an extremely high C/N value of 43, produced the overall lowest biogas. While the PM mono-digesters, that had a low C/N ratio of eight, also yielded low biogas, yet at a stable rate, with an average yield of 0.341 L/kg VS destroyed, in the first 30 days. However, this eventually picked up, and produced the overall highest average biogas production of 0.518 L/kg VS destroyed (see Table 2, and Fig. 5).

**Table 2:** C/N ratio and average biogas production for the different feedstocks.

**Feedstock**	**Components**	**C/N Ratio**	**Parts (%)**	**Final C/N Ratio**	**Average Biogas Production by Feedstock (L/kg VS destroyed)**
**PM Only**	Inoculate	15	20	8:1	0.5180
	PM	6	80		
**PM and KW**	Inoculate	15	20	11:1	Digester 3: 0.4175
	PM	6	60		Digester 4: 0.1944
	KW	20	20		
**PM and NPW**	Inoculate	15	20	43:1	0.2185
	PM	6	72		
	NPW	450	8		

### 3.2 Total and volatile solids

The total solids (TS) and volatile solids (VS) contents and the volatile solids reduced/destroyed (VSR) of the six feedstocks are shown under Table 3. Given the obtained values, all the digesters in the pilot-scale study underwent solid-state anaerobic digestion (SSAD) process. This can be accounted with the low water dilution of the feedstock, and the volume of inoculum added. Duan, Ran [12] recommended a substrate to inoculum ratio of 1.5 for PM mono-digestion, while the present study used a ratio of 4:1 for all its feedstock, i.e., 20 % of the active slurry volume.

**Table 3:** VSS results summary for all digesters.

Digester	Feedstock Type	Total Solids (TS)	Volatile Solids (VS)	VSR (%)
		Initial (%)	Initial (%)	
1	PM	49.68	94.83	22.80
2	PM	52.05	97.05	44.31
3	PM and KW	43.90	96.59	76.16
4	PM and KW	48.45	99.84	93.83
5	PM and NPW	23.04	99.66	97.42
6	PM and NPW	31.02	99.15	73.25

The high VS content readings in the initial feedstock, taken just before the starting of the digestion period, could explain the low biogas volumes produced compared to previous studies [9,13,19]. However, these studies also employ low VS percentages in order to reduce ammonia inhibition that arises with the high nitrogen present in PM; Since it has been shown that as substrate concentration increases, so does total ammoniacal nitrogen (TAN) concentrations [12]. This could explain the lowest values of VS destroyed in this study by the mono-digested feedstocks, consequently leading to the lag in biogas production. Farrow [14] also observed higher VS destruction from the co-digested substrates over the mono-digested feedstocks, consequently, accounting for the higher biogas yield by the co-digested substrate. However, in the present study, the biogas volume for the mono-digested PM feedstock eventually picked up after 30 days (see Fig. 5), and even accounted for the highest biogas production on a given day at 1.75 L/kg VS destroyed (see Table 4). Similar trends have also been observed in other studies [9,26], whereby due to an increase in HRT, the anaerobes were able to eventually acclimatize to the SSAD environment and enhance biogas production, proving the feasibility of SSAD.

**Table 4:** Summary of biogas and methane yield results of all digesters during its digestion process.

Digester	Average Biogas Production (L/kg VS destroyed)	Highest Biogas Production (L/kg VS destroyed)	Highest Methane Content (%)	Average Methane Production (L/kg VS destroyed)	Highest Methane Production (L/kg VS destroyed)
1	0.6491	1.754	65.1	0.3582	1.1351
2	0.3869	1.032	67.0	0.1659	0.5488
3	0.4175	0.9754	65.9	0.2319	0.6311
4	0.1944	0.4872	71.9	0.1184	0.2698
5	0.1857	0.4692	67.7	0.1074	0.2740
6	0.2514	0.6241	65.6	0.1481	0.3382

Despite the equivalent water dilution ratio carried out by the previous studies [27], the results from the present study prove that PM inherently have high TS values, in comparison to other livestock manure. Typically, as TS concentration decreases, particularly in PM feedstocks, biogas yield increases [14,22,28]. However, this was contradicted in the present study with the co-digested substrates of PM with NPW, that had the lowest initial TS concentration, yet still produced the overall lowest biogas volumes (see Table 4). This could be explained by the high lignin content present in NPW, which is known to be recalcitrant in nature under anaerobic conditions; Hence, this almost non-biodegradable property would have led to the low biogas and methane volumes produced [29]. Therefore, Ripley [30] concluded that feed composition has a more significant effect on VS destroyed than feed concentration, and incorporating equations of waste-specific constant for the operating variables of an anaerobic digester would be more relevant over VS degradation. This would be a reasonable method for PM because it is very heterogenous in nature, and its properties depend on multiple factors, such as animal feed, the environment that the birds are in, and the species the poultry birds belong to. Moreover, AD itself is a “dynamic process influenced by several parameters” [12]; For example, substrate and inoculum compositions, HRT, organic loading rate (OLR), temperature, pH and others.

### 3.3 Effects of temperature variation on biogas production

Out of all the operational parameters, the digester temperature seemed to have the most effect on biogas yield. Throughout the AD process, the environmental temperature had drastic changes at times, which also had an impact on digester temperatures. The variability of the digester temperatures is shown under Fig. 3. It should be noted that the pilot study was conducted in the summer season of Fiji in 2020 (November–April), which is infamously known as the “cyclone season” of the country. Consequently, in the duration of this study, two major tropical cyclones (TC) occurred, namely TC Yasa, which was a severe category five cyclone and TC Ana, which was a category three cyclone. The district had also encountered a few low-pressure systems during the pilot study, which otherwise is known to have an average of 233 annual sunshine days [31]. Hence, while these weather patterns could have been a recipe for disaster, it enabled the formation of valuable results in a ‘worst-case scenario’ situation. It is also noteworthy that the digesters were able to withstand the storms and suffer almost no damage. However, these extreme conditions did influence the optimal functioning of the anaerobic bacteria.

The gaps in substrate temperature readings in Fig. 3 are due to the extreme weather patterns that occurred during this time, which also caused the formation of scum layers in the digesters. Any major changes in weather patterns that resulted in temperature drops (or “temperature shock”), would consequently lead to a decrease in biogas yield. Similar trends have been reported by Babaee, Shayegan [9], Farrow [14], and Sakar, Yetilmezsoy [27], and is aptly explained by Babaee, Shayegan [9], whereby, “lower temperatures during the process are known to decrease microbial growth, substrate utilization rates, and biogas production. Moreover, a lower temperature may also result in an exhaustion of cell energy, a leakage of intracellular substances or complete lysis.” Furthermore, Fig. 3 indicates that when temperatures fell from 29 °C to 26 °C, biogas production decreased by up to 80 % for digester one. However, the reduction of biogas yield was not as drastic as in the co-digested feedstocks; For the same temperature drop, the biogas production for the simultaneously occurring digester three fell by 20 % only. Similarly, Babaee, Shayegan [9] observed a 50 % decrease in methane production when temperatures decreased by almost 10 °C during the co-digestion of PM and straw.

Additionally, Fig. 3 displays that the biogas yield from the mono-digested feedstock displayed more erratic patterns with every temperature change. The graph also shows the lag in stabilization of biogas yield after a period of severe weather. Even after a storm passes, and temperatures rose again, these sudden changes can also lead to reduced biogas production, which was also observed by Sakar, Yetilmezsoy [27]. Additionally, Fig. 3 also shows that the co-digested feedstock of KW had a more stable biogas yield, despite the drastic changes in temperature. This indicates that the anaerobes present in the co-digested feedstock were more resilient to temperature shocks, than those present in the mono-digested feedstock. Nevertheless, for optimal AD performance, the anaerobes need a more stable and controlled environment that will prevent temperature shocks of even 1 °C [14]. Therefore, it would be advantageous to install insulators in the anaerobic reactors.

### 3.4 pH influence on biogas yield

**Fig. 4:**
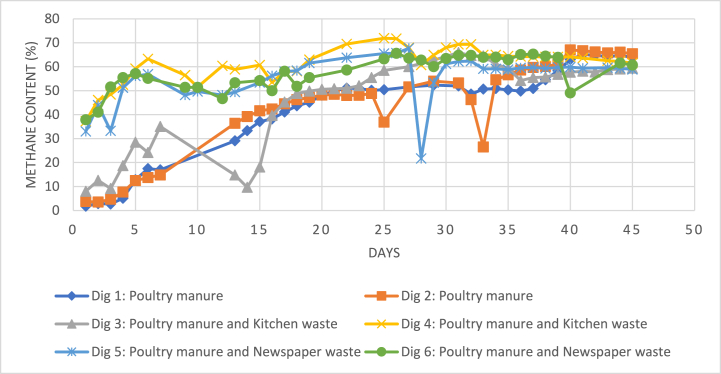
Methane percentage composition variation of all digesters during the digestion period.

The first batch of digesters (digesters 1, 2 and 3) began the AD process with pH values between 5.8 and 6. Gradually, these values started decreasing to a low of 5.6, indicating that the digesters were in the acid-forming stages, i.e., in the hydrolysis and acidogenesis stages. These initial values correspond to the literature values of 5.5–6.5 for the acid-forming period [8]. After 13–15 days, a rise in pH was observed in the three digesters, denoting that the reactors had entered its acetogenesis and methanogenesis phases. The rise in methane production during this period also verifies this observation (see Fig. 4), and agrees with previous studies, whereby increase in acetate degradation brings rise to pH and also, methane yield [13]. The pH then stabilized after 25 days at almost a neutral pH of 7, indicating a balance between the acid-forming and acid-consuming bacteria species [12,17].

**Fig. 5:**
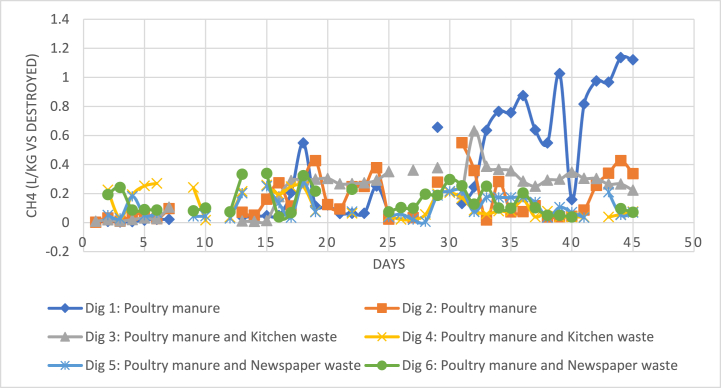
Methane volume yield variation of all digesters during the AD process.

Conversely, the second batch of the pilot AD study (digesters 4, 5 and 6) had an initial pH value of 6.6. The higher pH values in its initial stages could have been caused by the change in inoculum. Half of the inoculum for the second batch were obtained from the effluents of the mono-digesters in the first batch. These effluents already had anaerobes present and were collected when its PM feedstocks were showing rise in methane production (see Fig. 5). This would also explain the higher methane composition in digesters 4, 5 and 6, starting at 33–37 % and increasing to more than 60 % in just five days (see Fig. 4). However, despite the ‘healthy’ values of pH, the mono-digesters (digesters 1 and 2), still had lower methane production in comparison to the co-digested feedstock of KW in digester 3. Duan, Ran [12] explained this trend, whereby a TAN concentration above 3 g/L would still induce ammonia toxicity, irrespective of the digester pH.

### 3.5 Effect of co-digestion on biogas and methane yield

During the digestion period, the most consistent biogas and methane production was from the co-digested PM and KW feedstock (see Fig. 3, Fig. 5), with an average of 0.417 L/kg VS destroyed (see Table 4). However, due to the elevated levels of free ammonia present, the mono-digesters underwent a lag phase, leading to the late acclimatization of the anaerobes in the feedstocks. After the first 30 days, the mono-digesters eventually had the highest average biogas production of 0.649 L/kg VS destroyed, and digesters 1 and 2 also generated the overall highest biogas volumes in a day, during the studied 45-days digestion period (see Fig. 5 and Table 4). The PM feedstock was still producing biogas in the first 30 days, but at a very low rate. Lazor, Hutnan [28] describe this period as a “pseudo-stable” state, whereby the biogas yield is usually lower than optimum levels due to partial acclimatization of the bacteria consortium to the environment and hence, less than ideal bacterial functions.

However, in the case of inconsistent weather patterns, the co-digesters seemed to recover quicker to a stable state in comparison to the mono-digesters. It appears that the anaerobes in the co-digesters were more resilient to sudden changes in environment conditions. For instance, Fig. 3 clearly shows the correlation between biogas production and changes in temperature for digester 1, whereby both the variables seemed directly proportional. In contrast, the graph in Fig. 3 also shows a clear distinction of the biogas production in digester 3 from digester 1, whereby the biogas production remained more stable throughout the digestion period, despite the changes in temperature.

### 3.6 Possibility of inoculum influence on biogas production

It must be noted that in the first batch of the pilot study, for digesters 1, 2 and 3, 20 % of the total active reactor volume comprised of the cow slurry inoculum. This was essential to kickstart the digestion process as the cow manure contains the bacteria necessary for anaerobic digestion, which is not naturally found in poultry manure [32]. Therefore, the cow slurry inoculum could have contributed to an uncertain portion of the biogas produced. A limitation of the pilot study is that a control was not performed of the AD of the inoculum only. Hence, the exact amount of biogas produced that could be contributed by the inoculum is not known. However, the digesters in the second batch, had only half of its inoculum comprised of cow slurry, whilst the other half was obtained from the effluent of the mono-digesters in the first batch. Thus, making up only 10 % of the entire active volume, the cow slurry has a lesser influence on the biogas produced in the second batch.

### 3.7 Other operational observations and issues

All the digesters had encountered foaming and scum formation issues. Scum layers have been known to cause the development of temperature gradients within a digester, creating an inefficient environment for anaerobes and hence, lower than ideal biogas yield [33]. The layers were mostly formed after a period of severe weather and would be 10–15 cm thick. This was moderated by agitating the floating digester tank, i.e., rotating it clockwise and counterclockwise for a few minutes daily or when the scum layer has been formed.

The NPW feedstocks seemed to have the worst scum layer of approximately 15 cm, which could have been due to the hard cell wall structure of lignin. The foaming also made it problematic to take readings since the active slurry became inaccessible for the measuring probes. The low biogas readings during these periods may have also been caused by the scum layer which might have prevented the biogas from escaping the active slurry, and thus, stopping the floater tanks to rise.

Furthermore, the only damage that the digesters encountered during the severe tropical cyclones were the removal of the aluminium foil tapes that temporarily covered the holes for inserting the measuring probes. Due to the intense winds and wet weather, the tapes were partially removed, hence, causing the biogas to escape. This would also cause the floater tanks to collapse, contributing to the low biogas readings taken after the storms. The high and erratic oxygen readings observed during these periods also correlates well with this.

### 3.8 Full scale design

Taking into consideration the results obtained from the pilot study, a completely stirred tank reactor (CSTR) is proposed for the full-scale design of the AD system of poultry manure for the studied poultry farm. Installing a mixing rotor would combat the bulk of the issues that were encountered during the pilot study. It would ensure that the nutrient in the feedstock is distributed uniformly for the microorganisms. Additionally, both the accumulation of solids at the bottom of the digester tank, and the formation of scum layer at the top would be avoided. The mixing of the substrate would also release entrapped biogas in the slurry and therefore, increase the overall efficiency of the digester system, resulting in higher biogas yield [34]. Additionally, instead of a batch scheme that was adopted in the pilot study, a continuous loading system is proposed for the full-scale design, whereby feedstock would be loaded daily. A continuous loading mechanism incorporated with a CSTR has proven to be more efficient, and can produce up to four times more methane per reactor volume in comparison with batch AD systems [35].

The results of the mono-digesters from the pilot scale study were used to design the proposed full scale AD system, and Table 5 presents a summary of the vital factors of the design, including the operational parameters and calculated potential outputs. An HRT of 35 days has been chosen, which is ideal for a CSTR at a mesophilic temperature range [8]. This provides an active reactor volume of 95.78 m^3^ and would normally account to 75 % of the total digester volume (V_T_), thus producing an actual total bioreactor volume of V_T_ = 130 m^3^. Also, considering the HRT, the presumed volume of the storage tank is 50 m^3^, where the digestate would be temporarily stored before being sold as an organic liquid fertilizer.

**Table 5:** Full scale design summary of principal parameters.

**Operational Parameters**	
Available daily feedstock	912.20 kg/day
Substrate flow rate, Q	2736.60 L/day
Active reactor volume	95.78 m^3^
Total digester volume, V_T_	130 m^3^
Substrate inflow, S	162.67 kg VS/m^3^
Organic loading rate, OLR	4.65 kg VS/m^3^ reactor volume
**Calculated daily outputs**	
Biogas production, Q_biogas_	288.38 m^3^/day
Energy output, E_biogas_	1.73 MWh
Useable electrical energy, E_electricity_	519.08 KWh
Heat energy, E_heat_	951.65 KWh

The calculated reactor volume is on a smaller scale in comparison to a wet or semi-solid AD approach. SSAD has the advantages of employing lower reactor volumes, and thus, reduced energy required for heating, less water for dilution and, less wastewater or effluent produced [13]. However, with SSAD of PM there are risks of high ammonia levels and employing longer HRTs. Yet, the results from the pilot study have proven that despite being in a dry AD environment, the anaerobes are eventually able to acclimatize to these conditions, and even produce biogas volumes comparable to the other co-digested feedstocks.

The calculated organic loading rate (OLR) has a favourable value of 4.65 kgVS/m^3^ reactor volume, as it falls within the recommended OLR range of 4–8 kgVS/m^3^, for a CSTR with a continuously loading AD system [8]. Therefore, the digester system would have a healthy and suitable environment for the anaerobic bacteria consortium to function well and would not be overloaded with the mass of volatile solids feedstocks being filled daily, which could lead to digester failure.

The calculated biogas production for the full-scale design was obtained by approximating a minimum increase of 25 % in biogas yield from the results of the mono-digested feedstocks in the pilot study. This has been done to account for the installation of a mixing rotor, as studies have proven that biogas production can grow by more than 100 % with a mixing regime in comparison with unmixed substrates [34]. As a result, with the daily biogas production, the proposed full-scale design has a potential to produce 105,258.7 m^3^ of biogas annually.

The actual useable daily electricity production was derived by taking into account the electrical efficiency of a combined heat and power (CHP) biogas engine, which is usually equivalent to 30 % [8]. Therefore, the poultry farm has an annual total electricity production potential of 189.46 MWh. The daily electricity production is sufficient to meet the needs of both the digester system, and the poultry farm, whilst still produce a daily surplus of 510.64 KWh. Therefore, the proposed full scale biodigester system is also technically feasible.

Furthermore, considering the default emission factors from the IPCC *2006 Guidelines for National Greenhouse Gas Inventories* [36], the proposed full scale bioreactor would reduce a total of 55.98 tCO_2eq._ emissions every year.

A 5-kW biogas-fuelled, CHP engine is proposed to power both the full-scale digester, and the poultry farm. The heat energy for the full-scale design is recommended to be applied for heating the effluent rather than the digester. An insulation material is suggested for the biodigester to maintain its constant temperature to combat the fluctuating biogas yield that was encountered during the pilot study. Out of the possible total energy that could be produced by the biogas (see Table 5), a 55 % efficiency is taken into account for the heat energy generated by the CHP engine [8]. Hence, the total heat energy of 951.654 KWh would be able to raise the temperature of the effluent by 20.48 °C, assuming the temperature of the feedstock is consistent due to the applications of the CSTR and insulation material.

### 3.9 Economic feasibility

The summary of the financial costs of the full-scale design is shown under Table 6. The price for phenolic resin insulation is also included under “hidden cost”, with a recommended insulation thickness of 0.09 m which will be used to maintain an unchanging temperature of 35 °C [37]. The total recurring benefits include the revenue that would be generated by selling the daily surplus electricity generated to the national grid at a local independent power producers (IPP) tariff rate of FJD 0.33 per KWh [38]. Additionally, it includes the annual savings that the poultry farm would make in electricity consumption within the expected lifetime of the proposed project of 20 years.

**Table 6:** Financial considerations of the proposed full-scale design.

Items	Costs (FJD)
**Capital Costs**	
130 m^3^ digester + 50 m^3^ storage tank	488,750.00
5 kW CHP Biogas engine	34,650.00
Hidden costs	50,000.00
10 % contingency	57,340.00
**Total capital cost**	**630,740.00**
**Recurring Costs**	
Maintenance	5000.00
Operator	20,000.00
**Total annual costs**	**25,000.00**
**Recurring Benefits**	
IPP tariff revenue	61,506.59
Annual savings in electricity bill	1077.72
**Total recurring benefits**	**62,584.31**

The entire digester system can be paid off in under 17 years, which is less than the expected lifetime of the project. The LCOE of the full-scale system is FJD 0.17/KWh, which is almost half the value of Fiji's IPP tariff rate at FJD 0.33/KWh. Notably, the LCOE calculated for the proposed application is almost equivalent to the global average LCOE of biomass energy at FJD 0.16/KWh [39]. Thus, the proposed full scale AD system design for the studied poultry farm is also economically feasible.

### 3.10 Effluent application: fertilizer benefit

The effluent or digestate by-product of the AD process is rich in nutrients like nitrogen, potassium and phosphorus, which can act as a great fertilizer [40]. For the full-scale design, the remaining heat energy generated by the CHP engine is proposed to be used for heating the storage tank containing the effluent instead of the biodigester. Even with the possible heat loss during the transfer of the effluent from the biodigester to the storage tank, the results show that the temperature of the effluent can rise to more than 50 °C with the heat supplied from the CHP engine. This can be favourable for the quality of the effluent utilized as a biofertilizer. The mesophilic temperature range of AD has been known to reduce significant levels of pathogens present in the digestate; Whilst studies have also shown that the higher the effluent temperature, the greater the pathogenic destruction [9]. This would make the digestate much safer in its application on soils as a biofertilizer or soil conditioner.

Furthermore, selling the effluent as a fertilizer increases the financial profitability of the proposed full scale biodigester system. Suppose even 5 % of the annual digestate produced is sold as biofertilizer at the current market price of approximately FJD 20.00 for each 5 L bottle [41], the total annual benefits would increase by approximately FJD 200,000.00, and significantly reduce the SPB from 16.8 years to just under 3 years.

### 3.11 Biogas as clean cooking fuel

The average consumption for a biogas cooking stove is 0.4 m^3^ biogas/hour [8]. While the recorded highest number of hours spent cooking in a week by consumers was 13.2 h, which is approximately 2 h a day [42]. Hence, the annual biogas production by the farm of 105,258.7 m^3^, could provide 263,146.75 h of cooking annually, which would be sufficient to cater more than 383 households for an entire year. Moreover, the annual biogas production has the capacity to fill more than 3759 domestic LPG cylinders [43]. The calorific value of biogas, however, is significantly lower to that of LPG, which could range from 6 to 6.5 KWh/m^3^ [8]. Nevertheless, the proposed AD system would be able to reduce yearly LPG consumption by 48,867 kg, which would reduce CO_2_ emissions by 147.09 tonnes annually [44]. In comparison with using the biogas for electricity generation, the GHG reduction is significantly higher as there is no major apparent loss in energy when using biogas as cooking fuel. However, the approximate 55 % of energy that would be converted to heat by the CHP engine has not been accounted for in the GHG reduction as it is not used originally in the base case [8].

## 4 Conclusion

In this work, out of all the operational parameters, fluctuations in temperature had the greatest effect on biogas production for all feedstocks. The co-digested feedstocks of KW and NPW with PM appeared to be more resilient to changes in environmental conditions over the mono-digested substrate of PM. This study proves that the proposed full-scale system of the SSAD of PM, for the studied poultry farm, is both technically and financially feasible. It has the potential to produce 189.46 MWh of electricity annually, whilst reducing 55.98 tCO_2eq._ in GHG emissions. The system also has a SPB of 17 years, and a LCOE of FJD 0.17/kWh. Future works could focus on a comparison study between feedstocks of different total solids content, and to determine if the economic benefits of high TS content would outweigh the higher methane production from lower TS content.

## Funding

This research was funded by the *School of Information Technology, Engineering, Mathematics and Physics, of the University of the South Pacific**.*

## Data availability

Datasets related to this article can be found at https://doi.org/10.6073/pasta/dc2cd8a6544775834ea21c94de40baef, an open-source online data repository hosted at *Environmental Data Initiative* [45].

## CRediT authorship contribution statement

**Geeta M. Naidu:** Writing – original draft, Methodology, Investigation, Formal analysis, Data curation, Conceptualization. **Atul Raturi:** Writing – review & editing, Supervision, Software, Conceptualization. **Francis S. Mani:** Writing – review & editing, Supervision, Resources.

## Declaration of competing interest

The authors declare the following financial interests/personal relationships which may be considered as potential competing interests: The owner of the poultry farm in study is a parent of the corresponding author.

## References

[1] United Nations (2022). World population to reach 8 billion on 15 November 2022. https://www.un.org/en/desa/world-population-reach-8-billion-15-november-2022.

[2] S&P Global Platts (2021). https://www.spglobal.com/platts/en/market-insights/latest-news/oil/100621-global-energy-demand-to-grow-47-by-2050-with-oil-still-top-source-us-eia.

[3] United Nations (2021). Goal 7:affordable and clean energy. https://www.un.org/development/desa/disabilities/envision2030-goal7.html.

[4] (2021). Irena. Energy Profile Fiji.

[5] Cole S.W., S, Quigley S. (2019).

[6] Poultry J.a.H. (2021).

[7] AL-Huqail A.A. (2022). Sustainable valorization of four types of fruit peel waste for biogas recovery and use of digestate for radish (raphanus sativus L. Cv. Pusa himani) cultivation. Sustainability.

[8] Vögeli Y. (2014).

[9] Babaee A., Shayegan J., Roshani A. (2013). Anaerobic slurry co-digestion of poultry manure and straw: effect of organic loading and temperature. Journal of environmental health science & engineering.

[10] (2022). NEXSTEP. Fiji.

[11] IRENA (2015).

[12] Duan N. (2018). Performance evaluation of mesophilic anaerobic digestion of chicken manure with algal digestate. Energies.

[13] Abouelenien F. (2016). Dry Co-digestion of poultry manure with agriculture wastes. Appl. Biochem. Biotechnol..

[14] Farrow C. (2016).

[15] Shapovalov Y. (2020). Dry anaerobic digestion of chicken manure: a review. Appl. Sci..

[16] DVO (2022). Poultry litter digestion. https://dvoinc.com/poultry.php.

[17] Hilborn V. (2011).

[18] Boysan F. (2015). Biogas production from animal manure. Procedia Earth and Planetary Science.

[19] Carlini M., Castellucci S., Moneti M. (2015). Biogas production from poultry manure and cheese whey wastewater under mesophilic conditions in batch reactor. Energy Proc..

[20] (2001). Hanna instruments. HI 8314.

[21] Greer T. (2021). Carbon to nitrogen compost calculator: create the perfect compost pile. https://morningchores.com/compost-calculator/.

[22] Dahunsi S. (2019). Co-digestion of Theobroma cacao (Cocoa) pod husk and poultry manure for energy generation: effects of pretreatment methods. Bioresour. Technol..

[23] U.S. Environmental Protection Agency (2001). Method 1684 total, fixed, and volatile solids in water. Solids, and Biosolids.

[24] de Oliveira Paranhos A.G. (2020). Methane production by co-digestion of poultry manure and lignocellulosic biomass: kinetic and energy assessment. Bioresour. Technol..

[25] Ribeiro E.M. (2018). Feasibility of biogas and energy generation from poultry manure in Brazil. Waste Manag. Res..

[26] Hills D.J., Ravishanker P. (1984). Methane gas from high solids digestion of poultry manure and wheat straw. Poultry Sci..

[27] Sakar S., Yetilmezsoy K., Kocak E. (2009). Anaerobic digestion technology in poultry and livestock waste treatment — a literature review. Waste Manag. Res..

[28] Lazor M. (2010). 37th International Conference of SSCHE May..

[29] Gonzalez-Estrella J. (2017). A review of anaerobic digestion of paper and paper board waste. Rev. Environ. Sci. Biotechnol..

[30] Ripley L.E. (1988).

[31] Fiji Meteorological Service (2021). Fiji meteorological service. https://www.met.gov.fj/index.php?page=index_smartmet.

[32] Sims J.T., Maguire R.O., Hillel D. (2005). Encyclopedia of Soils in the Environment.

[33] Shiyou P. (2019). A novel hydraulic biogas digester controlling the scum formation in batch and semi-continuous tests using banana stems. Bioresour. Technol..

[34] Singh B., Szamosi Z., Siménfalvi Z. (2020). Impact of mixing intensity and duration on biogas production in an anaerobic digester: a review. Crit. Rev. Biotechnol..

[35] Igoni A. (2008). Comparative evaluation of batch and continuous anaerobic digesters in biogas production from municipal solid waste using mathematical models. Agric. Eng. Int. CIGR J..

[36] Eggleton H.S., Miwa K., Ngara T., Tanabe K., IPCC (2006). IPCC Guidelines for National Greenhouse Gas Inventories, B.L..

[37] Guo P. (2019). Biogas production and heat transfer performance of a multiphase flow digester. Energies.

[38] Fiji Department of Energy (2014).

[39] Renewable I.R.E.N.A. (2021). Power generation costs in 2020. https://www.irena.org/-/media/Files/IRENA/Agency/Publication/2021/Jun/IRENA_Power_Generation_Costs_2020.pdf.

[40] Dornelas K.C., Schneider R.M., Do Amaral A.G. (2017). Biogas from poultry waste—production and energy potential. Environ. Monit. Assess..

[41] OrganicPlus Fiji (2020). Organic liquid fertilizer. https://www.facebook.com/organicplusfiji/.

[42] Statista Research Department (2015). Time spent cooking per week worldwide 2014, by country. https://www.statista.com/statistics/420719/time-spent-cooking-per-week-among-consumers-by-country/.

[43] GPS Renewables (2022). What and why of biogas.

[44] The Engineering Toolbox (2009). Combustion of fuels-carbon dioxide emission. https://www.engineeringtoolbox.com/co2-emission-fuels-d_1085.html.

[45] Naidu G.M. (2023). Ba: Techno-Economic Study.

